# Maternal Undernutrition Effect on Pregnancy-Associated Glycoprotein (PAG) Concentration in Sheep Carrying Single and Multiple Fetuses

**DOI:** 10.3390/ani14233427

**Published:** 2024-11-27

**Authors:** Olimpia Barbato, Vittoria Lucia Barile, Laura Menchetti, Giovanni Ricci, Emilia Larisa Achihaei, Cristian Porcu, Francesca Daniela Sotgiu, Valeria Pasciu, Fiammetta Berlinguer

**Affiliations:** 1 Department of Veterinary Medicine, University of Perugia, Via San Costanzo 4, 06126 Perugia, Italy; olimpia.barbato@unipg.it (O.B.); giovanni.ricci@unipg.it (G.R.); larisa.achi@gmail.com (E.L.A.); 2 Research Centre for Animal Production and Aquaculture, Consiglio per la Ricerca in Agricoltura e l’Analisi dell’Economia Agraria (CREA), Via Salaria 31, 00015 Monterotondo, Italy; vittorialucia.barile@crea.gov.it; 3 School of Biosciences and Veterinary Medicine, University of Camerino, Via Circonvallazione 93/95, 62024 Matelica, Italy; 4 Department of Veterinary Medicine, University of Sassari, Via Vienna 2, 07100 Sassari, Italy; porcu.cristian@gmail.com (C.P.); fdsotgiu@uniss.it (F.D.S.); vpasciu@uniss.it (V.P.); berling@uniss.it (F.B.)

**Keywords:** sheep, PAGs, single pregnancy, multiple pregnancy, undernourishment

## Abstract

To the best of our knowledge, no studies in the literature have reported on the relationship between the pregnancy-associated glycoprotein (PAG) concentration and undernutrition during pregnancy in sheep. Therefore, the aim of this study was to investigate the effects of undernutrition on the blood concentration of PAGs in pregnant ewes carrying single and multiple fetuses, undergoing either dietary energy restriction or receiving 100% of their energy requirements during the period of maximal placenta growth. From d 24 to 100 of pregnancy, the ewes were fed ryegrass hay and two different iso-proteic concentrates, fulfilling either 100% of the ewes’ energy requirements (control group; n = 30, 14 singleton pregnancies, 16 multiple pregnancies) or only 50% (feed-restricted group; n = 29; 11 singleton pregnancies, 18 multiple pregnancies). Blood samples were collected from all the ewes the day before intravaginal sponge insertion (T0) and, thereafter, every 30 days starting from d 24 of gestation (T1) until d 30 after parturition (T6), to determine the PAG, progesterone, NEFA, total protein, and glucose concentrations. Our results showed that PAG concentrations are not only influenced by the number of fetuses, but also by the ewe’s diet. In particular, the PAG marginal means were higher in the feed-restricted group than in the control group (7.8 ± 0.6 and 10.8 ± 0.9 ng/mL for the control and feed-restricted groups, respectively; *p* = 0.040). This finding confirms that a dietary restriction between d 24 and d 100 of gestation leads to a growth in placental functions as inferred from the increase in the concentration of the PAG levels. Therefore, the measurement of PAG concentrations, in addition to serving as a marker of pregnancy, can also serve as a marker of placental functionality.

## Simple Summary

The placenta serves as the vital interface between maternal and fetal circulation, playing a pivotal role in ensuring the fetus’s nutrition and oxygenation. It is, in fact, a highly sophisticated and multifaceted organ, capable of integrating signals from both the mother and the fetus with remarkable efficiency. PAG levels are indicative of the number of fetal cotyledons in the placenta and, consequently, the size of the placenta. This provides insight into placental development and fetal–placental well-being. However, to the best of our knowledge, the effect of maternal undernutrition on PAG secretion has not been extensively investigated in ruminant species. Starting from this premise, this study aimed to determine whether undernutrition might exert an influence on the production of this glycoprotein, with subsequent implications for embryonic and fetal well-being.

## 1. Introduction

The placenta serves as the vital interface between maternal and fetal circulation, playing a pivotal role in ensuring the fetus’s nutrition and oxygenation. It is, in fact, a highly sophisticated and multifaceted organ, capable of integrating signals from both the mother and the fetus with remarkable efficiency [1]. During pregnancy, the placenta undergoes a variety of physiological changes, regulated by angiogenic factors, hormones, and nutrient-related genes, in order to optimize efficiency and meet the ever-increasing demand for nutrients. Consequently, alterations in maternal nutrition during pregnancy can exert considerable influence on placental function and fetal development [2].

Several studies have been conducted to investigate the impact of maternal diet during distinct phases of pregnancy on placental and fetal development in sheep [3,4,5]. In sheep, placental growth is completed by d 100 of gestation and most fetal growth occurs by d 109 and thereafter [6]. Nutrient restriction over the initial 40 days or the final 50 days of pregnancy typically has no discernible impact on placental weight [2,7]. Conversely, the majority of studies examining the influence of a restricted diet over variable periods between 30 and 107 days of pregnancy have identified a significant modification in the placental weight [8,9,10]. Although the consequence of maternal undernutrition on placental weight is evident, the timing, duration, and cause of nutritional restriction each exert a distinct influence on placental mass [11].

In sheep, maternal undernutrition during the early to mid-pregnancy period resulted in an increased placental weight-to-fetal weight ratio at term, without any change in the fetal weight [9]. Conversely, in rats, it has been demonstrated that maternal undernutrition during the second half of pregnancy results in a reduction in the placental weight-to-fetal weight ratio [10]. This suggests that feed restriction has a long-term impact on placental weight, particularly when nutrient deprivation coincides with the period when fetal nutrient demand is at its highest.

It is important to consider the various functions performed by the placenta, including its impact on fetal growth and development through metabolic and endocrine mechanisms. These mechanisms include the secretion of pregnancy-associated glycoproteins (PAGs) and other glycoproteins into the maternal circulation by placental binucleate cells in ruminant species [12,13,14,15,16]. These proteins modulate fetal growth by stimulating the redistribution of maternal nutrients to the fetus. Furthermore, their concentrations in maternal circulation can be used as indicators of placental development and fetal–placental well-being, playing a key role in the maintenance of pregnancy in ruminant species [17,18,19].

The structure and function of the placenta can be altered by gestational under- or overnutrition, which in turn affects the transport efficiency of essential nutrients to the fetus at the trophoblast layer within the placentomes, where nutrient and gas exchange occurs [20,21]. The concentration of PAGs in the maternal circulation can be increased or decreased as a result of gestational undernutrition [22] or overnutrition [23], respectively.

Ovine PAGs are glycoproteins with a molecular weight ranging from 43 to 70 kDa and with a pI ranging from 4 to 6.8 [15,24]. In ovine placentas, 11 types of cDNA have been identified, each coding for a distinct PAG (ovPAG-1 to ovPAG-11) and at different specific gestational periods. This confirmed the multiplicity and temporal expression of PAG molecules in ruminant placentas [15,25]. The application of the RIA and ELISA methodologies has revealed that these molecules can be detected in ovine plasma as early as the 18th–20th day of pregnancy [24,26,27,28,29,30,31].

In this species, the concentrations of PAGs vary throughout pregnancy, in accordance with the breed, number of fetuses, sex, and birth weight [32,33]. In contrast to the pattern observed in other ruminant species [19,34], the profile of PAGs during gestation is biphasic. A pre-calving peak is not evident and the concentration reaches basal levels within four weeks postpartum. This difference is attributed to the behavior of binucleated cells, which in sheep have been observed to decrease in number [12,35].

PAG levels are indicative of the number of fetal cotyledons in the placenta and, consequently, the size of the placenta. They provide insight into placental development and fetal–placental well-being. However, to the best of our knowledge, the effect of maternal undernutrition on PAG secretion has not been extensively investigated in ruminant species. Starting from this premise, this study aimed to determine whether undernutrition might exert an influence on the production of these glycoproteins, with subsequent implications for embryonic and fetal well-being, in sheep with single or multiple pregnancies.

## 2. Materials and Methods

### 2.1. Ethics Statement

The experimental procedures involving animals (sheep, Ovis aries) were approved by the Animal Care and Use Committee at the University of Sassari and AGRIS, Italy. All the experimental work was carried out at the facilities of AGRIS (Bonassai, Sardegna, Italy). These facilities meet the requirements of the European Union for Scientific Procedure Establishments. The experimental procedures followed ethical guidelines on the care and use of animals for research (European Union Directive 2010/63/UE for animal experiments).

### 2.2. Animals and Experimental Procedures

This research is part of a study aimed at determining the modification in maternal circulation in terms of some metabolites’ key regulators of Nitric oxide, in undernourished sheep carrying single and multiple fetuses [36].

This study was carried out on 59 adults (5.0 ± 3.0 years) and multiparous pregnant ewes (Sarda breed) from the experimental flock of AGRIS Sardegna (Sassari, Italy). Pregnancy occurred after natural breeding, following cycle synchronization with intravaginal sponges impregnated with progestogens (20 mg of fluorogestone acetate, Chronogest© CR; MSD-AH, Madison, NJ, USA) for 12 days, plus a single i.m. injection of 200 IU of eCG (Folligon©; MSD-AH, MSD Animal Health, Milano, Italy), concurrent with pessary removal. At d 24 after the mating, pregnancy diagnosis and initial fetal counts were conducted by transrectal ultrasonography, with a real-time B-mode scanner (Aloka SSD 500; Aloka Co., Tokyo, Japan), fitted with a 7.5 MHz linear-array probe. After scanning, the ewes were pair matched into two groups (control and feed restricted), according to their age, body weight (BW), and number of fetuses (control group: BW 47.8 ± 5.2; n = 30, 14 singleton pregnancies and 16 multiple pregnancies; feed-restricted group: BW 48.1 ± 7.7; n = 29; 11 singleton pregnancies and 18 multiple pregnancies). At d 45, the fetal counts were reassessed, as previously described.

From d 24 to d 100 of pregnancy, the ewes were fed the experimental diets, as reported by [36], which consisted of ryegrass hay and two different iso-proteic concentrates (high and low energy concentrates), whose formula was aimed at either fulfilling the ewes’ energy requirements for maintenance and pregnancy (control group) or only 50% of their energy requirements (feed-restricted group), using the Small Ruminant Nutrition System [37]. The diets were fed at the level of 0.9 kg/ewe per day (50% energy requirement) and 1.5 kg/ewe per day (100% energy requirement). From d 100 to lambing, the experimental diets were adjusted fortnightly in order to account for the increasing requirements of pregnancy and the gap between the diets was progressively reduced from 50 to 40% in order to ensure adequate lamb viability at lambing.

### 2.3. Blood Sampling, Body Weight, and Body Condition Score

Blood samples were collected from all the ewes the day before intravaginal sponge insertion (T0) and, thereafter, every 30 days starting from d 24 of gestation until d 30 after parturition (T1 = d 24, T2 = d 50, T3 = d 80, T4 = d 110, T5 = d 140, T6 = d 30 postpartum). The blood was sampled at 07.00 a.m. (before morning meal administration), using 10 mL vacuum collection tubes containing EDTA K2 (Vacutainer Systems Europe; Becton Dickinson, Meylan Cedex, France), to determine the metabolic and hormonal profile (NEFA, total protein, progesterone, and PAGs); 3 mL vacuum collection tubes, containing lithium heparin and mono-iodoacetate (Vacutainer Systems Europe; Becton Dickinson, Meylan Cedex, France), were used to determine the glucose level. Immediately after collection, the blood samples were cooled at 4 °C and centrifuged at 1500× *g* for 15 min. The plasma was removed and stored at −20 °C, until analysis.

Throughout the experiment from T0 to T5, on the same days as the blood sampling, the maternal body condition and the body weight (BW) of the ewes were recorded and used to determine the body weight change during the gestational period. The body condition score (BCS) was assessed by the same operator using a 5-point scale, with 1 being emaciated and 5 being obese [38].

### 2.4. Assessment of Metabolic and Hormonal Profile

Glucose and NEFA were measured using commercial kits and a BS-200 Mindray clinical chemistry analyzer (NEFA: Wako Chemicals GMBT, Neuss, Germany. Glucose: Realtime Hagen Diagnostic Systems, Firenze, Italy; BS 200: Chemistry Analizer, Mindray, Shenzhen, China), as previously described [39]. The glucose concentrations were determined in a single assay using the liquid enzymatic colorimetric method (GOD-POD) (real-time kit), with a glucose standard of 100 mg/dL for calibration. The intra-assay CV values were 1.1%. The NEFA concentrations were measured in multiple assays using the enzymatic endpoint method (Diagnostic Systems kits). The NEFA intra-assay and inter-assay CV values were 1.07% and 0.98%, respectively. The plasma protein content was measured using Lowry’s method.

The progesterone concentration was measured in duplicate using a commercial ELISA kit (DRG Instruments GmbH, Marburg, Germany), which is a solid-phase ELISA, based on the principle of competitive binding. The ELISA assay was performed using the Personal Lab by Adaltis (Adaltis srl, Rome, Italy), which is a tool that performs automated ELISA protocols. The analytical sensitivity was 0.045 ng/mL and the intra-assay and inter-assay CV values were <10%.

The PAG concentrations were determined by RIA-srPool assays, previously described by [27]. Pure boPAG_67kDa_ preparation was used as the standard and tracer. Iodination (Na-I^125^, Amersham Pharmacia Biotech, Uppsala, Sweden) was carried out according to the chloramine-T method, previously described by [40]. The samples were assayed in a preincubated system, according to which the standard curve ranged from 0.2 to 25 ng/mL. The intra- and inter-assay coefficients were 2.5% and 7.5%, respectively.

### 2.5. Statistical Analysis

Our study employed comprehensive statistical analysis. Diagnostic charts were used to verify the assumptions and identify outliers. The PAG and P4 concentrations were log (x + 1) transformed, but the raw data are presented here. The between-group differences, the effect of the number of lambs born, and the changes over time, were analyzed using linear mixed models, including the time as repeated (assuming an exchangeable working correlation matrix) and the animals as random factors. The models evaluated the effects of the group (2 levels: control and feed-restricted diet), time (6 levels, from d 24-T1 to postpartum-T6), the number of lambs born (categorized into two levels: single and multiple lambs), and their interactions. The values before nutritional treatment (T0) were included as a covariate and expressed as parameter b (±standard error, SE). The Šidák correction was used for multiple comparisons [41]. Furthermore, to better understand and visualize the effect of the interactions between nutrition and the number of lambs on the analyzed parameters, at each time point, multiple comparisons between the following four subgroups were performed and presented: control diet and single lamb, control diet and multiple lambs, feed-restricted diet and single lamb, and feed-restricted diet and multiple lambs.

Pearson’s correlation coefficient (r) was used to evaluate the correlation between indicators of the body condition, hormones, and metabolites at each time point. A poor correlation was considered if r < |0.3|, a medium correlation if |0.3| ≤ r < |0.5|, and a large correlation if r ≥ |0.5.| [42]. This analysis provided insights into the relationships between these variables, regardless of the group.

Finally, in order to gain a multidimensional view of the variables during pregnancy, principal component analysis (PCA) was conducted with the data at d 110 (T4). The purpose of this analysis was to identify relationships among the variables and identify multivariable dimensions that could describe the main changes related to diet and prolificacy. The Kaiser–Meyer–Olkin (KMO) test was used to verify the adequacy of the sampling. For simplicity, the first two components (PCs) were retained, rotated using the varimax method, and interpreted. Only factor loadings with an absolute value greater than 0.4 were discussed [43]. The regression scores were calculated using the regression method.

The statistical analyses were performed using SPSS Statistics version 25 (IBM, SPSS Inc., Chicago, IL, USA). Statistical significance occurred when *p* < 0.05.

## 3. Results

At parturition, the number of single and multiple lambs recorded by ultrasonography was confirmed.

### 3.1. Body Weight and BCS

**Figure 1:**
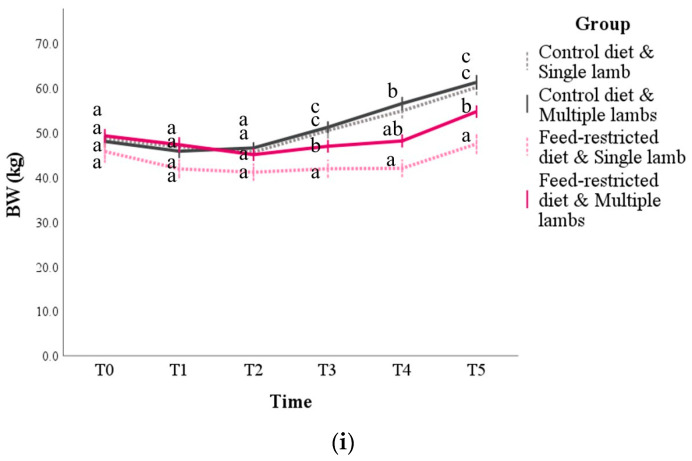

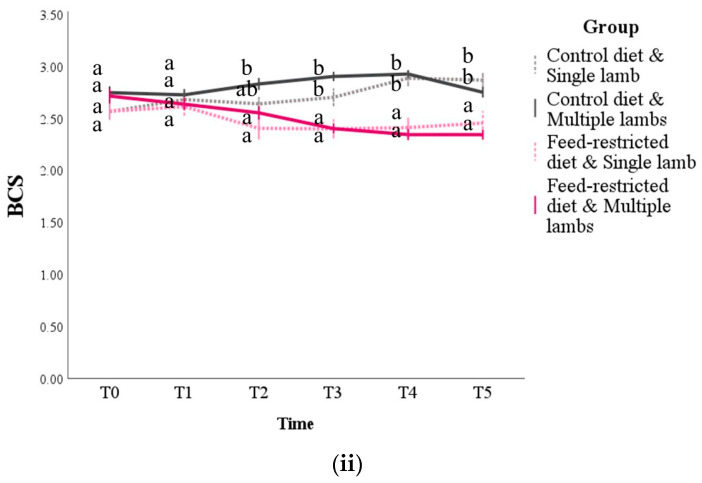
Body weight (BW, panel (**i**)) and body condition score (BCS, panel (**ii**)) during pregnancy and postpartum in sheep according to the diet (control or feed restricted) and the number of lambs born (one or more than one, respectively, defined as single and multiple lambs). T0 = 2 weeks before mating; T1 to T5 = day of pregnancy: T1 = d 24, T2 = d 50, T3 = d 80, T4 = d 110, and T5 = d 140. The values are the mean and standard error. For each time point, the mean of the groups that do not share a letter are statistically different (*p* < 0.05).

Body weight was influenced by all the main factors analyzed (time, diet, number of lambs, basal values), as well as by some interactions between the factors (*p* < 0.05; Supplementary Materials Table S1). In particular, body weight increased over time, but the increase was smaller in the feed-restricted (mean difference ± SE: −5.7 ± 0.9 kg; *p* < 0.001) and single-birth animals (mean difference ± SE: −5.7 ± 0.9 kg; *p* = 0.023). The pairwise comparisons highlighted that these differences started from time T3 (Figure 1i) when the feed-restricted and single-lamb animals had the lowest BW, followed by the feed-restricted and multiple lambs subgroup; the highest values, without differences due to the number of lambs, were found in the animals fed the control diet (*p* < 0.001). This trend was maintained until T5 (*p* < 0.01) and was comparable to what was found for the BCS (Figure 1ii and Supplementary Materials Table S1).

### 3.2. PAGs and Progesterone

**Figure 2:**
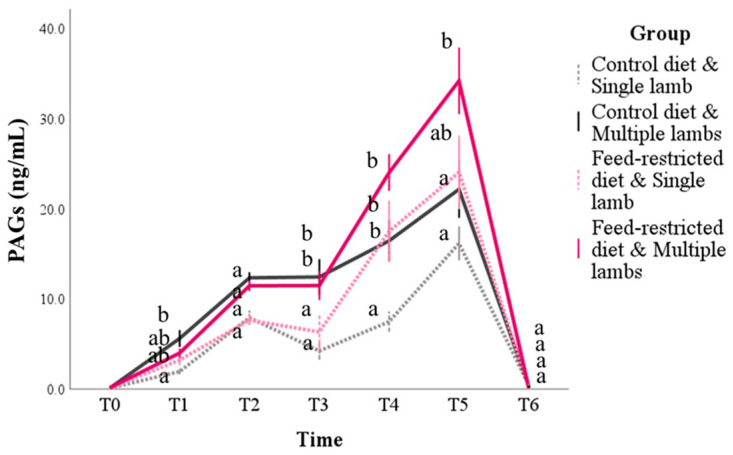
PAG concentrations during pregnancy and postpartum in sheep according to the diet (control or feed restricted) and the number of lambs born (one or more than one, respectively, defined as single and multiple lambs). T0 = 2 weeks before mating; T1 to T5 = day of pregnancy: T1 = d 24, T2 = d 50, T3 = d 80, T4 = d 110, T5 = d 140, and T6 = d30 postpartum. The values are the mean and standard error. For each time point, the mean of the groups that do not share a letter are statistically different (*p* < 0.05).

The PAG concentrations were influenced by the group (*p* = 0.040), time (*p* < 0.001), number of lambs (*p* < 0.001), and the interaction between the time and the group (*p* < 0.001) and between the time and the number of lambs (*p* < 0.001; Figure 2 and Supplementary Materials Table S1). Regardless of the group, the PAG concentrations progressively increased until they reached a peak at T5 (*p* < 0.001) and then fell sharply postpartum (at T6 *p* < 0.001). As regards the group effect, the animals in the feed-restricted group had higher marginal means than those in the control diet group (7.8 ± 0.6 and 10.8 ± 0.9 ng/mL for the control and feed-restricted groups, respectively; *p* = 0.040). The pairwise comparisons confirmed that the PAG levels in the feed-restricted group were higher than the control group from T1 to T4 (*p* < 0.01). The number of lambs influenced the PAG concentrations, since the marginal mean of the animals giving birth to more than one lamb was higher than those giving birth to a single lamb (6.8 ± 0.7 and 11.2 ± 0.8 ng/mL for single and multiple lamb groups, respectively; *p* < 0.001). Multiple comparisons between the subgroups further highlighted the interaction effect of nutrition, the number of lambs, and time (Figure 2). In particular, at T1, the control diet and single-lamb subgroup showed lower values than those of the control diet and multiple lambs subgroup (*p* = 0.002). At T3, all the animals that had given birth to a single lamb (control diet and single lamb and feed restricted and single lamb) had lower values than the multiple birth animals, regardless of nutrition (*p* < 0.001). At T4, the control diet and single-lamb subgroup had the lowest values, while there were no significant differences between the other groups. Finally, at T5, both the control diet subgroups (single and multiple lambs) had lower values than the feed-restricted and multiple lambs subgroup (*p* < 0.01), while the feed-restricted and single-lamb subgroup had intermediate values. At T5, in fact, the animals in the feed-restricted and multiple lambs group reached the highest peak in terms of PAG concentrations, with values above 30 ng/mL (34.2 ± 3.7 ng/mL).

**Figure 3:**
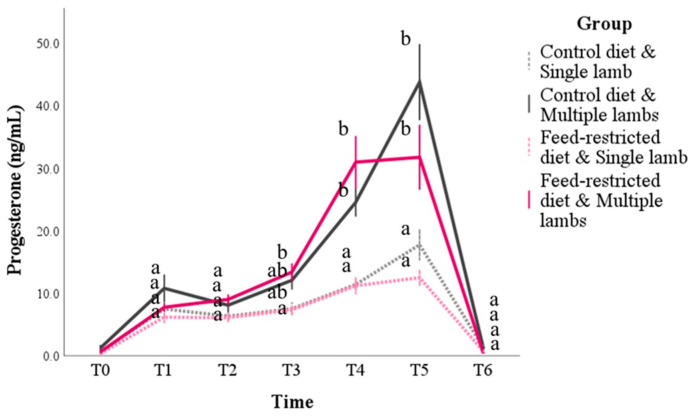
Progesterone concentrations during pregnancy and postpartum in sheep according to the diet (control or feed restricted) and the number of lambs born (one or more than one, respectively, defined as single and multiple lambs). T0 = 2 weeks before mating; T1 to T5 = day of pregnancy: T1 = d 24, T2 = d 50, T3 = d 80, T4 = d 110, T5 = d 140, and T6= d 30 postpartum. The values are the mean and standard error. For each time point, the mean of the groups that do not share a letter are statistically different (*p* < 0.05).

Progesterone was affected by time (*p* < 0.001), the number of lambs (*p* < 0.001), and the interaction between the time and the group (*p* = 0.005) and between the time and the number of lambs (*p* < 0.001), while the effect of the diet was not significant (Supplementary Materials Table S1). For all the animals, progesterone increased progressively until T5. However, from T3 to T5, the effect of the number of lambs was highlighted in multiple comparisons (Figure 3). At T3, the control diet and single-lamb subgroup showed lower progesterone levels than the feed-restricted and multiple lambs subgroup (*p* < 0.001), while at T4 and T5, regardless of the diet, all the animals that had given birth to a single lamb had lower progesterone values than the ewes with multiple births (*p* < 0.001).

### 3.3. Metabolites

**Figure 4:**
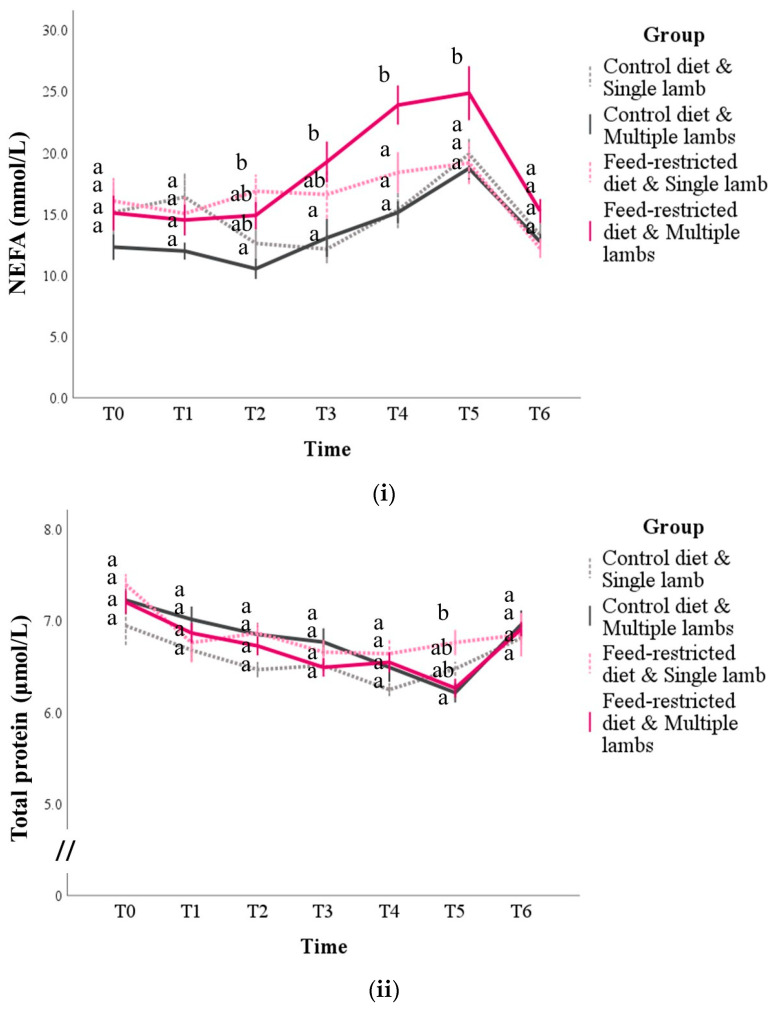

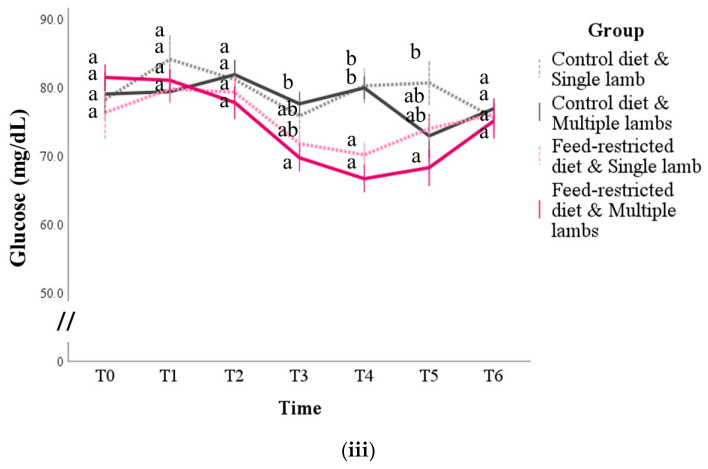
NEFA (panel (**i**)), total protein (panel (**ii**)), and glucose (panel (**iii**)) concentrations during pregnancy and postpartum in sheep according to the diet (control or feed restricted) and the number of lambs born (one or more than one, respectively, defined as single and multiple lambs). T0 = 2 weeks before mating; T1 to T5 = day of pregnancy: T1 = d 24, T2 = d 50, T3 = d 80, T4 = d 110, T5 = d 140, and T6 = d 30 postpartum. The values are the mean and standard error. For each time point, the mean of the groups that do not share a letter are statistically different (*p* < 0.05).

The NEFA concentrations were influenced by the time, group, and interaction between the time and the group and between the time and the number of lambs, in addition to their baseline levels (for all: *p* < 0.01; Supplementary Materials Table S1). Overall, the NEFA concentrations increased until T5, but the marginal means were higher in the feed-restricted group than in the control group (mean difference ± SE: +2.9 ± 0.9; *p* = 0.004), while the main effect of the number of lambs was not significant (*p* = 0.250). The pairwise comparisons showed that from T2 to T5, the NEFA concentrations in the feed-restricted group increased more consistently and, in particular, ewes in the feed-restricted and multiple lambs subgroup had the highest levels (*p* < 0.05; Figure 4i).

The protein concentrations were influenced by their basal levels (*p* < 0.001), time (*p* < 0.001), the time and group interaction (*p* = 0.045), and the time and number of lambs interaction (*p*< 0.001; Supplementary Materials Table S1). However, multiple comparisons were only able to find significant differences between the control diet and multiple lambs vs. the feed-restricted and single-lamb subgroups at T5 (*p* = 0.014; Figure 4ii).

The glucose concentrations, in addition to their basal levels (*p* = 0.003), were influenced by time (*p* < 0.001) and feed restriction (*p* = 0.005), while the only trend was found for the interaction between the time and the number of lambs (*p* = 0.053; Supplementary Materials Table S1). The diet had a predominant effect over that of the number of lambs, confirmed by multiple comparisons: at T3, the control diet and multiple lambs subgroup had a higher value than the feed-restricted diet and multiple lambs subgroup (*p* = 0.027); at T4, all the feed-restricted animals had lower concentrations than the animals given the control diet, regardless of the number of lambs (*p* < 0.001); finally, at T5, the control diet and single-lamb subgroup showed higher concentrations than the feed-restricted and multiple lambs subgroup (*p* < 0.001; Figure 4iii).

### 3.4. Correlations Between the Variables and Multivariable Analysis

Supplementary Materials Table S2 shows the correlations between the parameters analyzed for each time point, regardless of the group. From T2 to T4, the PAG concentrations were positively correlated with progesterone (*p* < 0.01), while they showed a positive correlation with NEFA only at T4. Furthermore, at T4, glucose was strongly positively correlated with BW and BCS, while showing a moderate negative correlation with the PAG and progesterone concerntrations (*p* < 0.01). Although weaker, progesterone maintained this correlation with the glucose concentration, even at T5 (*p* < 0.05).

**Figure 5:**
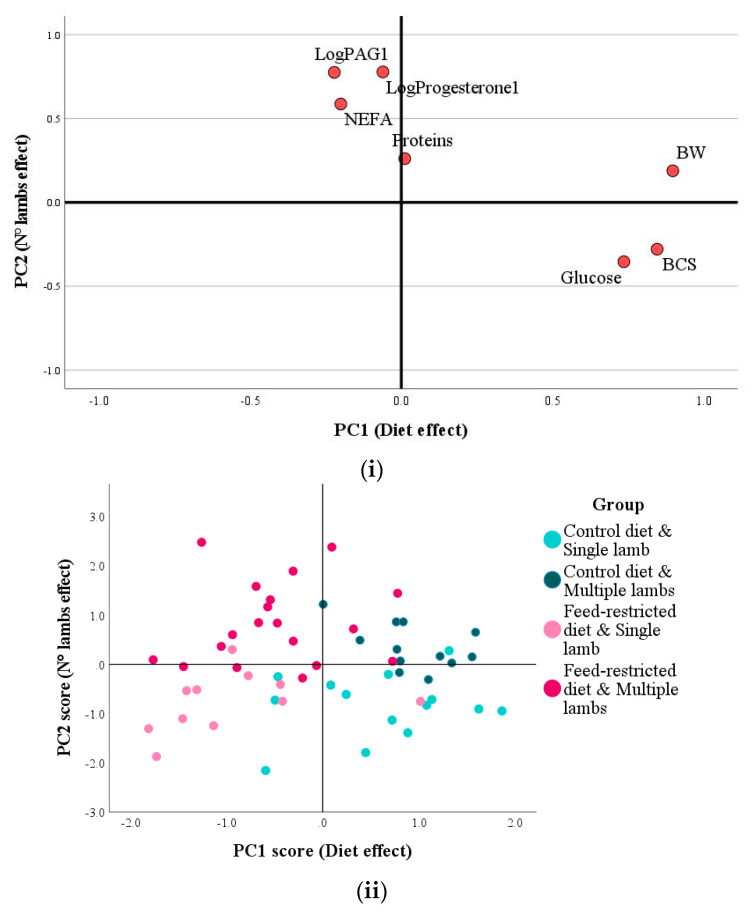
Principal component (PC) plot in rotated space indicating the loadings of the variables (panel (**i**)) and the PC scores of the animals (panel (**ii**)), where the colors of the dots indicate the group (i.e., control and feed-restricted diet) and the number of lambs born (one or more than one, respectively, defined as single and multiple lambs). The interpretation of the variable loadings suggested the labels “Diet effect” and “Number of lambs effect” for PC1 and PC2, respectively.

The relationships between the variables were further investigated with PCA, of which the KMO (=0.623) confirmed the sample size adequacy. The loadings of the variables in terms of the first two components, accounting for 57.2% of the total variance, are illustrated in Figure 5i and detailed in Supplementary Materials Table S3. PC1 (represented in the x-axis of Figure 5i) had high positive loadings for BW, BCS, and glucose concentration, while PC2 (y-axis) had high positive loading for PAG, progesterone, and NEFA concerntrations. Figure 5ii shows the PC scores according to the diet and number of lambs. The scores for the control diet group were concentrated in the right quadrants (I and IV), while those of the feed-restricted group were concentrated in the left one (II and III). On the contrary, the animals were arranged in the upper or lower quadrants mainly according to the number of lambs that they gave birth to, as follows: ewes that had given birth to a single lamb were mostly in the lower quadrants (III and IV), while ewes that had given birth to multiple lambs were in the upper ones (II and I). This arrangement indicates that PC1 mainly characterizes the effect of diet thanks to the variables BW, BCS, and glucose concentration. The effect of the number of lambs was linked to PC2 with variations, especially in regard to the progesterone, PAG, and NEFA concentrations. Thus, the interpretation of the variable loadings suggested the labels “Diet effect” and “Number of lambs effect” for PC1 and PC2, respectively. The protein loadings did not reach 0.4 in regard to these PCs, suggesting that they were not significantly involved in the changes due to diet or the number of lambs.

## 4. Discussion

Maternal nutrition is an important variable known to influence placental growth and birth weight in a variety of species [2,44]. However, only by gaining a thorough understanding of the actions of nutrition during pregnancy and placental function can important issues related to animal welfare and fetal development be fully understood.

In sheep, the evidence on the dietary effects in terms of changes in placental growth is inconclusive. Several studies have shown that overfeeding ewes during pregnancy results in a profound and consistent reduction in placental size, leading to a significant reduction in birth weight [9,23]. Other studies [8,23] have reported a significant reduction in the number of fetal cotyledons associated with high dietary intake during the first trimester; furthermore, their results indicated that high dietary intake during the second trimester had the most pronounced negative impact on placental growth and its capacity to transfer nutrients to the developing fetus. On the contrary, studies examining the effect of restricted diets for periods of varying length during pregnancy found an increase in the weight of the placenta [11,45].

To the best of our knowledge, no studies in the literature have reported on the relationship between PAG concentrations and undernutrition during pregnancy in sheep, except for the work of [46], which studies maternal diet manipulation during the three-week period before mating. These authors found that diet manipulation, either below or above the nutritional requirements, during the pre-mating period, did not affect the synthesis of PAGs or progesterone in either single or twin pregnancies. Therefore, this paper can be considered the first to address the influence of undernutrition during pregnancy on placental activity in sheep.

The results of our study showed that the PAG concentrations increased gradually until day 140 of gestation, after which they declined significantly during the postpartum period. Furthermore, our findings align with the bimodal profile observed in other sheep breeds, as documented by several authors [23,27,30,31,35], as a result of dynamic changes in placental function throughout gestation [13,23].

Although the PAGs trend remained unchanged during pregnancy, the PAG concentration values showed variations according to the diet utilized. In the feed-restricted group, the PAGs marginal means were higher than those of the control group, especially from d 80 of gestation. This suggests that a reduction in diet between d 24 and d 100 of gestation leads to growth in placental function. In sheep, placental growth has been reported to peak between d 75 and d 90 of gestation [2,47]. Thus, the significant increase in the PAG concentrations recorded in undernourished ewes could reflect the increase in the number of fetal cotyledons, providing an index on the placental secretory capacity.

In cattle, studies have demonstrated that placental function in heifers, as assessed by circulating concentrations of PL, PAG, P4, and ES, was enhanced by dietary restriction [22]. A reduction in dietary protein levels during the first trimester was associated with elevated PAG concentrations, indicating that a diet deficient in protein resulted in enhanced placental function at various stages of gestation. This phenomenon has been attributed to hormonal signalling by the fetus, indicating an increased need for nutrients [48].

The number of lambs influenced the PAG concentrations since the marginal mean of the animals giving birth to more than one lamb was higher than those giving birth to a single lamb. This finding was expected, given that higher PAG concentrations in pregnancies involving multiple fetuses are related to the increase in placental mass, corresponding to an increase in the number of binucleate giant cells, which are the main source of PAGs [30,35]. In our work, this difference starts to be significant at d 80 of pregnancy, which is the period corresponding to the placental growth in sheep, as previously discussed. In fact, all animals that gave birth to a single lamb had lower values than the multiple-birth animals, in both nutritional groups. Starting from d 80 of pregnancy, the feed-restricted diet and multiple lambs subgroup had higher PAG concentration values than the others. These findings confirm that maternal undernutrition determines an important stage of growth in the number of fetal cotyledons in the placenta, as reported by other authors [8,49], particularly in pregnancies involving multiple fetuses.

Similar to what was observed for PAGs, the progesterone concentration exhibited a constant increase throughout pregnancy, before declining after d 140, in all groups. This increase is more significant in multiple than in singleton pregnancies, according to the literature. It is acknowledged that the concentration of progesterone increases in proportion to the number of fetuses [32,50]. This observation lends support to the concept that progesterone plays a role in fetal growth [51] and that both the progesterone concentration and placental efficiency are influenced by the litter size [52]. While the time and the number of fetuses have an influence on progesterone levels, diet appears to have no significant effect. In agreement with previous works studying sheep [46,53] and other ruminants [54,55], a positive correlation was found between the PAG and progesterone concentrations, confirming that both are essential for embryo survival and a successful pregnancy.

As regards to metabolites, our study showed that dietary restriction based on a low-energy and iso-proteic diet had an effect on the circulating concentrations of NEFA and glucose, as expected, but no effect on those of total proteins.

The increase in the NEFA concentration in ewes during pregnancy indicates the mobilization of energy reserves, particularly during late gestation [56]. The energy derived from lipolysis promotes gluconeogenesis, thereby conserving glucose and amino acids for the growing fetus [56,57]. Accordingly, a moderate increase in the NEFA concentration during the late stages of pregnancy can be regarded as an adaptive mechanism for the partitioning of nutrients, while the energy balance at the onset of pregnancy is positive, as the requirements for fetal growth are relatively limited [58]. In agreement with what other authors have found [57,58], the NEFA concentration increase in our study was more pronounced in ewes subjected to food restriction during mid- and late gestation, indicating the critical mobilization of body reserves when food restriction occurs during the most energetically demanding phase of gestation.

The concentration of glucose also serves to reflect the physiological behaviors of this metabolite during pregnancy. Feed restriction affected the mean glucose plasma concentrations and had a predominant effect on the number of lambs carried. Ewe fetuses utilize a substantial amount of maternal glucose during mid- and late pregnancy [39,45,59]. In the event that nutritional necessities are not fulfilled during these stages, glucose homeostatic regulation is unable to maintain an equilibrium, resulting in a decline in the mean glucose level. In our study, the presence of low glucose concentrations and an elevated NEFA concentration is linked to the increased energy deficit observed in animals subjected to feed restriction during pregnancy.

As revealed by the PCA, our study showed that undernutrition and lamb numbers are positively correlated with PAG concentrations and, to a lesser extent, with progesterone and NEFA concentrations, while a negative correlation was confirmed with BW, BCS, and glucose levels. The multivariate analysis (i.e., PCA) confirms the results described above and also provides a “comprehensive picture” of the changes in all the parameters evaluated and represents how diet and the number of fetuses modulate and interact with these variables. Variations in the feed intake affect, as expected, impact the BCS and glucose concentration, as indicators of energy metabolism. Not only the NEFA concentration, but also PAG concentrations, move in the opposite direction. As a matter of fact, if food intake is restricted, while the body condition and circulating glucose are reduced, the PAG and NEFA concentrations increase. The effect of the number of fetuses are different but intertwined in modulating physiological responses. An increase in the fetal number results in an increase in the PAG, progesterone, and NEFA concentrations, while the changes in the BCS and glucose level go in the opposite direction (i.e., they decrease). These finely modulated changes could represent coordinated responses to ensure the mother’s energy homeostasis and fetal growth maintenance.

## 5. Conclusions

The increase in PAG concentrations in this study showed the influence of undernutrition on placental activity in sheep. It can be postulated that in conditions of undernutrition, the increased development of the placenta may represent a strategy to enhance the efficiency of maternal–fetal exchanges, thereby ensuring the optimal transfer of energy to the fetus. Therefore, the measurement of PAG concentrations, in addition to serving as a marker of pregnancy, can also serve as a marker of placental functionality.

## Data Availability

The original contributions presented in the study are included in the article/Supplementary Materials, further inquiries can be directed to the corresponding authors.

## Supplementary Materials

The following supporting information can be downloaded at: https://www.mdpi.com/article/10.3390/ani14233427/s1, Table S1: *p* values of the effects evaluated in the linear mixed models of all the variables examined; Table S2: Pearson correlation coefficient (r) between the analyzed parameters for each time point; Table S3: Principal components loadings, and variance. The interpretation of the variable loadings suggested the labels “Diet effect” and “Number of lambs effect” for the PC1 and PC2, respectively.
