# Supplementary material for: Maternal Undernutrition Effect on Pregnancy-Associated Glycoprotein (PAG) Concentration in Sheep Carrying Single and Multiple Fetuses
Source: Animals (Basel). 2024 Nov 27;14(23):3427. doi: 10.3390/ani14233427 (PMC11639935; doi:10.3390/ani14233427)
Supplement: Supplementary file 1 [file animals-14-03427-s001.zip › animals-3265182-supplementary.pdf]

Supplementary material

Table S1. P values of the effects evaluated in the linear mixed models of all the variables examined.

**Parameter**	**Group (Control vs Restricted diet)**	**Time**	**Number of lambs (single vs multiple lambs)**	**Group x Time**	**Group x Number of lambs**	**Number of lambs x Time**	**Group x Time x Number of lambs**	**Baseline values**
BW	<0.001	<0.001	0.023	<0.001	0.324	0.014	0.096	<0.001
BCS	0.043	<0.001	0.538	<0.001	0.528	<0.001	0.255	
PAGs	0.040	<0.001	<0.001	<0.001	0.293	<0.001	0.226	0.534
Progesterone	0.163	<0.001	<0.001	0.005	0.907	<0.001	0.721	0.508
Total proteins	0.713	<0.001	0.576	0.045	0.662	<0.001	0.454	<0.001
NEFA	0.004	<0.001	0.250	<0.001	0.092	<0.001	0.139	0.001
Glucose	0.005	<0.001	0.136	<0.001	0.634	0.053	0.297	0.003

Table S2. Pearson correlation coefficient (r) between the analyzed parameters for each time point.

Time		BCS	PAG[1]	Progesterone[1]	Protein	Triglycerides	Glucose
T0	BW	**.565****	.127	-.190	.042	.114	-.084
	BCS		.060	.050	.030	.030	.210
	PAGs[1]			-.012	-.206	-.160	-.151
	Progesterone[1]				-.066	-.040	.020
	Protein					.197	.239
	NEFA						.243
T1	BW	**.537****	.060	.196	-.015	**.287***	.236
	BCS		-.117	.144	.079	.230	**.437****
	PAGs[1]			.224	-.056	-.192	**-.298***
	Progesterone[1]				-.120	-.021	.113
	Protein					.120	-.037
	NEFA						.179
T2	BW	**.670****	.234	.031	.046	-.162	**.310***
	BCS		.206	.159	.109	-.237	**.488****
	PAGs[1]			**.428****	.126	-.115	-.040
	Progesterone[1]				-.004	-.050	.018
	Protein					-.008	.074
	NEFA						-.013
T3	BW	**.628****	.060	-.042	-.013	-.007	**.336****
	BCS		-.101	-.083	.074	**-.259***	**.367****
	PAGs[1]			**.449****	-.064	.119	-.086
	Progesterone[1]				-.060	.019	-.172
	Protein					-.029	.077
	NEFA						.118
T4	BW	**.638****	-.187	.100	-.047	-.117	**.503****
	BCS		**-.340****	**-.274***	-.111	**-.381****	**.637****
	PAGs[1]			**.455****	.161	**.411****	**-.382****
	Progesterone[1]				.003	.188	**-.404****
	Protein					.120	-.034
	NEFA						-.205
T5	BW	**.482****	-.199	.244	-.219	.102	.153
	BCS		-.246	-.262	.146	-.222	**.494****

	PAGs[1]			.137	-.058	.170	.017
	Progesterone[1]				**-.269***	.253	**-.330***
	Protein					-.031	.150
	NEFA						-.178
T6	PAGs[1]	-	-	.192	.012	-.051	.058
	Progesterone[1]				.049	-.171	.022
	Protein					.198	**.406****
	NEFA						.019

Significant r values are in bold: **. Correlation is significant at the 0.01 level (2-tailed). *. Correlation is significant at the 0.05 level (2-tailed).
1. Log transformed

Table S3. Principal components loadings, and variance. The interpretation of the variable loadings suggested the labels "Diet effect" and "Number of lambs effect" for the PC1 and PC2, respectively.

**Variables and parameters**	**PC1 (Diet effect)**	**PC2 (Number of lambs effect)**
**BW**	.904	.174
**BCS**	.832	-.276
**Glucose**	.718	-.445
**Progesterone**	-.025	.880
**PAGs**	-.187	.731
**Proteins**	.019	-.054
**Triglycerides**	-.175	.437
**% variance explained**	38.3%	19.0%
**Cumulative variance explained**	57.3%	
